# Supplementary material for: A small bacteriophage protein determines the hierarchy over co-residential jumbo phage in Bacillus thuringiensis serovar israelensis
Source: Commun Biol. 2022 Nov 24;5:1286. doi: 10.1038/s42003-022-04238-3 (PMC9700832; doi:10.1038/s42003-022-04238-3)
Supplement: Supplementary file 2 — Supplemementary Information [file 42003_2022_4238_MOESM2_ESM.pdf]

Supplementary Material

A small bacteriophage protein determines the hierarchy over co-residential jumbo phage in *Bacillus thuringiensis* serovar *israelensis*

Anja Pavlin[1,#], Anže Lovše[1,2,#], Gregor Bajc[1], Jan Otoničar[1], Amela Kujović[1], Živa Lengar[3], Ion Gutierrez-Aguirre[3], Rok Kostanjšek[1], Janez Konc[4], Nadine Fornelos[5], Matej Butala[1]

[1] Department of Biology, Biotechnical Faculty, University of Ljubljana, 1000 Ljubljana, Slovenia.

[2] Genialis, Inc., Boston, MA, USA.

[3] Department of Biotechnology and Systems Biology, National Institute of Biology, 1000 Ljubljana, Slovenia.

[4] Theory Department, National Institute of Chemistry, 1000 Ljubljana, Slovenia.

[5] Broad Institute of MIT and Harvard, Cambridge, MA, USA.

Supplementary Methods

Supplementary Figures 1-10

Supplementary Tables 1-2

Supplementary Methods

Bacterial cell lysate preparation and LexA affinity purification

Cultures of *S. aureus* ATCC 29213 and *E. coli* MG1655 were grown aerobically at 37°C in 300 mL of LB, to an OD_{600} of 1. Cells were harvested by centrifugation (15 min, 6000× *g*, 4°C), and cell pellets were resuspended (10 mL per 1 g pellet) in an extraction buffer (50 mM Tris-HCl, pH 7.5, 100 mM NaCl, 10% sucrose, 2 mM $MgCl_2$, 0.1% [v/v] Triton X-100) containing a protease-inhibitor cocktail tablet (Roche Diagnostics), 1 U mL^{-1} benzonase nuclease, 10 µg mL^{-1} RNAse A, 0.2 mg mL^{-1} lysozyme, and additionally for lysis of *S. aureus* cells, 0.01 mg mL^{-1} lysostaphin (AMBI Products LC). The mixture was incubated at 37°C for 1 h, cooled on ice, and sonicated three times for 30 s. The samples were then centrifuged (18000× *g*, 20 min, 4°C) to obtain clear lysates. Protein pulldowns were performed using Ni-chelate chromatography (Qiagen). Purified LexA proteins from *S. aureus* and *E. coli* (both carrying an N-terminal hexahistidine tag) were prepared as previously described [1], loaded onto pre-equilibrated columns and incubated for 30 min at 4°C. Excess LexA was washed off with wash buffer (50 mM NaH_2PO_4, pH 8.0, 300 mM NaCl, 20 mM imidazole). The *S. aureus* and *E. coli* cleared cell lysates were applied to beads immobilized with their respective LexA proteins, incubated at 4°C for 30 min, and then washed extensively with wash buffer. LexA interactors were eluted in 1.5 mL of elution buffer (50 mM NaH_2PO_4, pH 8.0, 300 mM NaCl, 250 mM imidazole) and concentrated by trichloroacetic acid precipitation. Proteins were resolved on 4% to 12% Bis-Tris protein gels (NuPAGE; Invitrogen) and stained with SimplyBlue SafeStain (Invitrogen). For protein identification, gel slices were excised and analyzed using a mass spectrometer (Thermo-Finnigan LTQ Orbitrap) at the Functional Genomics, Proteomics and Metabolomics Facility, University of Birmingham, Birmingham, UK.

Reverse transcription and quantitative PCR (qPCR)

To measure the native gp7 expression levels after activation of the lytic cycle of GIL01, we compared the gp7 levels in the untreated GBJ002(GIL01) strain and in the same strain 30 min after the MMC treatment. To assess the extent of ectopic gp7 expression from the pDG7 plasmid, we then compared the native gp7 levels with the levels detected in the GBJ002(pDG7) strain 30 minutes after the MMC induction. Total RNA was extracted from strains GBJ002(GIL01) and GBJ002(pDG7) as described in the main methods. Three biological replicates were prepared per strain and growth condition. Oligonucleotides for RT-qPCR (Table S8), used to multiply the gp7 and the housekeeping gene *tufA,* were designed in a previous study [2]. 200-400 ng of total RNA was reverse-transcribed into cDNA in a 20 µl reaction mixture using the High-Capacity cDNA Reverse Transcription Kit (Applied Biosystems™) according to the manufacturer's instructions with the addition of RNase inhibitor

(Applied Biosystems™) supplied separately. qPCR reactions were performed in 10 µL of reaction volume using the PowerUp™ SYBR™ Green Master Mix (Applied Biosystems), 600 nM of the appropriate forward and reverse primers, and 2 µL of cDNA according to the manufacturer's instructions. PCR was performed in 384-well plates (Applied Biosystems). Reactions were performed in triplicate on an ABI PRISM 7900HT Sequence Detection System (Applied Biosystems). The quantification cycle (Cq) for each amplification was determined using SDS 2.4 software (Applied Biosystems). For all calculations, the baseline was set automatically and the fluorescence threshold was set manually. Non-template controls were used to monitor for possible contamination of the qPCR reagents. Relative expression analysis of the qPCR results was performed using quantGenius software [3]. Relative copy numbers of the gp7 cDNA were normalized to the housekeeping gene tufA.

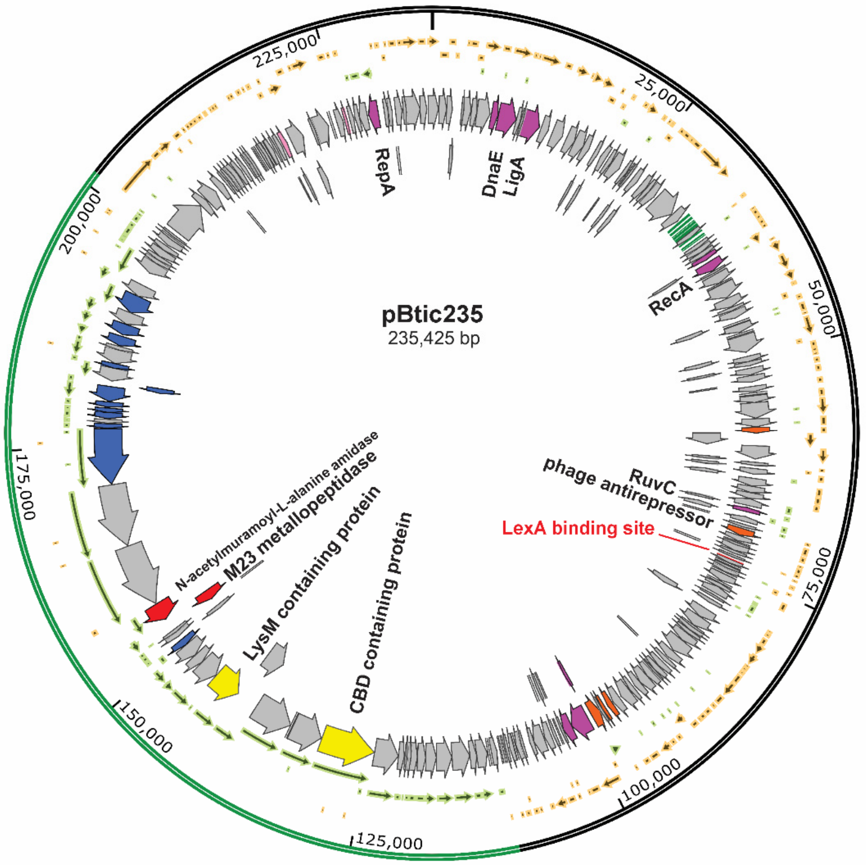

Supplementary Figure 1. Map of the pBtic235 genome. Putative ORFs are shown as arrows in the innermost edge of the circle. Arrow colors indicate different gene functions: blue for structural, red for lysis, yellow for host recognition, purple for DNA replication, orange for regulation, green for tRNA, and gray for uncharacterized. Selected relevant gene names and the LexA binding site are also indicated. The middle arrow line represents putative ORFs on the sense (orange) and anti-sense (green) strands. The outermost line denotes the plasmid (black) and phage (green) modules. Adapted from [4].

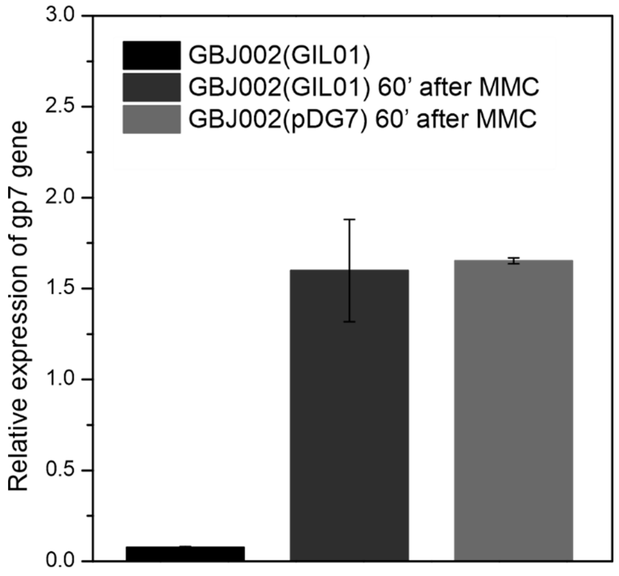

Supplementary Figure 2. Gp7 gene transcription from the IPTG-induced pDG7 overexpression plasmid or from the GIL01 prophage is comparable in SOS conditions. Transcript levels were determined by quantitative PCR in the GBJ002 strain carrying either the pDG7 or GIL01 prophage. Where indicated, 100 ng mL^{-1} MMC was added to the cultures for 1 h. To induce gp7 expression from pDG7, IPTG (0.1 mM) was added ~2 h before the MMC addition. Presented is the expression of the gp7 gene, relative to the expression from the housekeeping gene *tufA*. The data shown are the average of three biological experiments with three replicates and include the standard deviations.

a

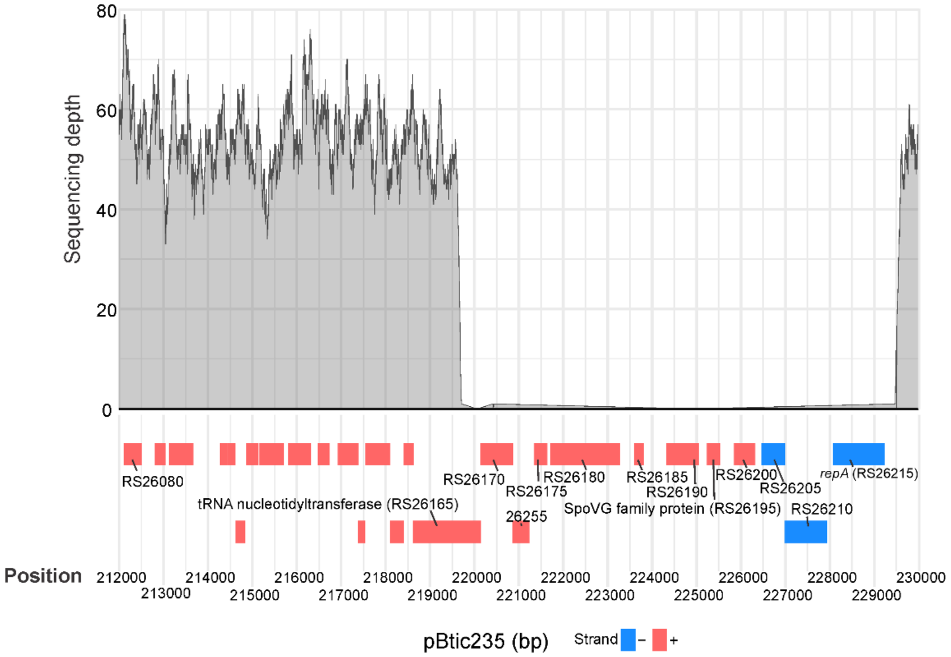

b

Gene ID	Protein ID	Aminoacid sequence length	NCBI homology based annotation	InterPro protein domain search
HIS92_RS26165	WP_000424042.1	507	tRNA nucleotidyltransferase	NA
HIS92_RS26170	WP_001071098.1	242	Hypothetical phage protein	None predicted
HIS92_26225	WP_000589206.1	51	Hypothetical protein	None predicted
HIS92_RS26175	WP_000502605.1	97	Hypothetical protein	None predicted
HIS92_RS26180	WP_000108739.1	524	Hypothetical protein	None predicted
HIS92_RS26185	WP_000149726.1	72	Hypothetical protein	None predicted
HIS92_RS26190	WP_000229935.1	241	Hypothetical protein	None predicted
HIS92_RS26195	WP_000455658.1	99	SpoVG family protein	NA
HIS92_RS26200	WP_000783539.1	155	Hypothetical protein	None predicted
HIS92_RS26205	WP_001097001.1	174	Hypothetical protein	None predicted
HIS92_RS26210	WP_000174333.1	313	Hypothetical protein	Actin-like ATPase domain
HIS92_RS26215	WP_000039126.1	383	Replication initiator protein A	NA

*NA (Not Assessed)

Supplementary Figure 3. The deleted region of the pBtic235 clear-plaque variant. a Sequencing depth of the deleted genomic region and its flanking regions (number of reads matching to the region from 212 to 230 kb) in the clear-plaque pBtic235 variant. Gene ID numbers correspond to the new locus tags, except for the old locus tag for gene “26255”, which is not annotated in the reference genome sequence (GenBank ID, NZ_CP051859). pBtic235 sequencing depth (read coverage) is shown in gray. The pBtic235 genes encoded on sense and anti-sense strands are given in red and blue, respectively. **b** List of deleted genes in the clear-plaque pBtic235 variant.

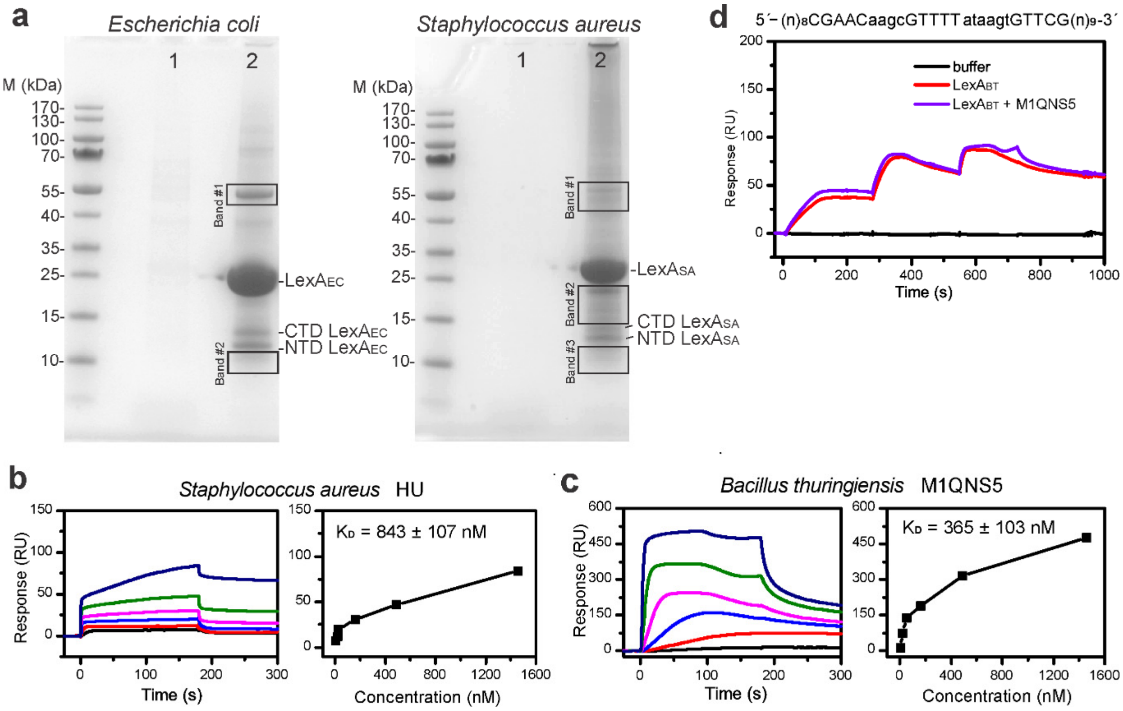

Supplementary Figure 4. Identification of *Escherichia coli* and *Staphylococcus aureus* LexA interactors. a Coomassie stained protein profile of affinity chromatography eluates from the empty affinity resin (lanes 1) and LexA (lanes 2, $LexA_{EC}$ from *E. coli*, left; $LexA_{SA}$ from *S. aureus*, right). Boxed proteins were digested with trypsin and analysed by mass spectrometry (Supplementary Data 6). Interactors that met the criteria of a molecular weight <10 kDa and ≥50% sequence coverage in mass spectrometry analysis only included the nucleoid-associated protein HU of *S. aureus*. Bands corresponding to intact LexA or self-cleaved repressor (CTD, NTD) are indicated to the right of both gels, and protein standards (M) are indicated to the left. **b** SPR sensorgrams of the interaction of the purified *S. aureus* HU protein with chip-immobilized $LexA_{SA}$. *S. aureus* HU and $LexA_{SA}$ formed a moderate affinity complex with an apparent equilibrium dissociation constant (K_D) of 843 nM, which indicated that the interaction was not biologically relevant. **c** The ProBiS algorithm was used to search the SWISS-MODEL Repository for gp7-like proteins of *E. coli*, *S. aureus*, and *B. thuringiensis* that consist of 40-100 amino acids and that share surface structural features with those of gp7. No protein from *S. aureus* or *E. coli* met the criteria of the analysis threshold. Among the *B. thuringiensis* proteins, the highest Z-score of 2.4 was assigned to the uncharacterized protein M1QNS5, which consists of 49 amino-acid residues. SPR sensorgrams of the interaction of the purified *B. thuringiensis* proteins M1QNS5 and *B. thuringiensis* $LexA_{BT}$. Purified M1QNS5 formed a complex with $LexA_{BT}$, with a K_D of 365 nM. **b, c** HU and M1QNS5 proteins were injected at three-fold dilutions (6-1458 nM) for 180 s at a flow rate of 30 µL min^{-1} over chip-immobilized LexA. Apparent equilibrium dissociation constants (K_D) are expressed as means ± standard deviation of two titrations of each

analyte. **d** SPR sensorgrams of the interaction between $LexA_{BT}$ alone and pre-incubated with M1QNS5 with the LexA operator of the GIL01 *P1* promoter region. The DNA sequence used is shown above the diagrams, with important nucleotides for LexA binding shown in capital letters. Free $LexA_{BT}$ (5, 10, 20 nM) or $LexA_{BT}$ (5, 10, 20 nM) pre-incubated with M1QNS5 in 1:10 molar ratio (50, 100, 200 nM) was injected over the chip-immobilized DNA at 50 µL min^{-1} in a single cycle. The experiment was performed in duplicate, and a representative sensorgram is shown. M1QNS5 protein did not increase the affinity of $LexA_{BT}$ for its target DNA sequence. Thus, these data suggested that M1QNS5 might affect the LexA function in a different manner to that of gp7.

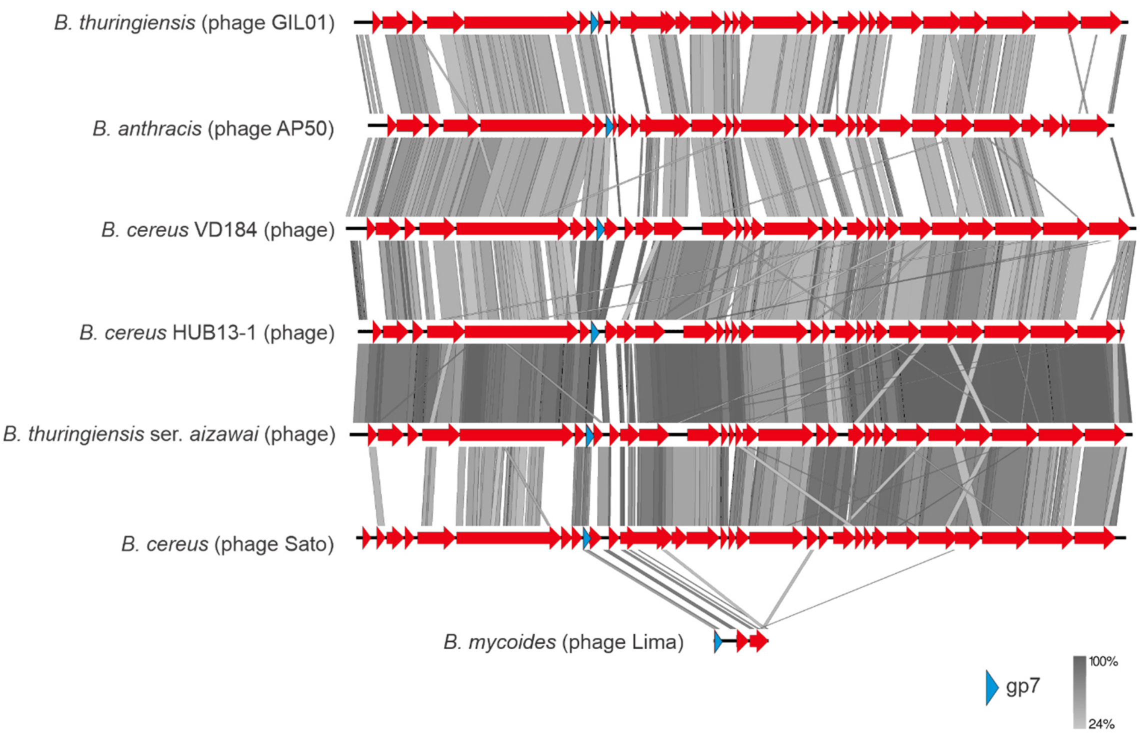

Supplementary Figure 5. **Alignment of tectivirus genomes.** Genome alignment of tectivirus genomes: *B. thuringiensis* phage GIL01 (GenBank ID: AJ536073.2) *Bacillus cereus* VD184 phage (GenBank ID: NZ_KB976851.1), *B. thuringiensis* serovar *aizawai* phage (GenBank ID: NZ_AMXT02000219.1), *B. cereus* HuB13-1 phage (GenBank ID: KB976635.1), *Bacillus mycoides* VDm034 phage Lima (GenBank ID: KC152964.1) *Bacillus anthracis* phage AP50 (GenBank ID, EU408779.1), *B. cereus* AND1284 phage Sato (GenBank ID, KC152965.1) was performed and visualized with Easyfig using tblastx [5]. The position of gp7 in the different tectivirus genomes is shown with a blue arrowhead, and the respective hosts are indicated to the left of the prophage maps.

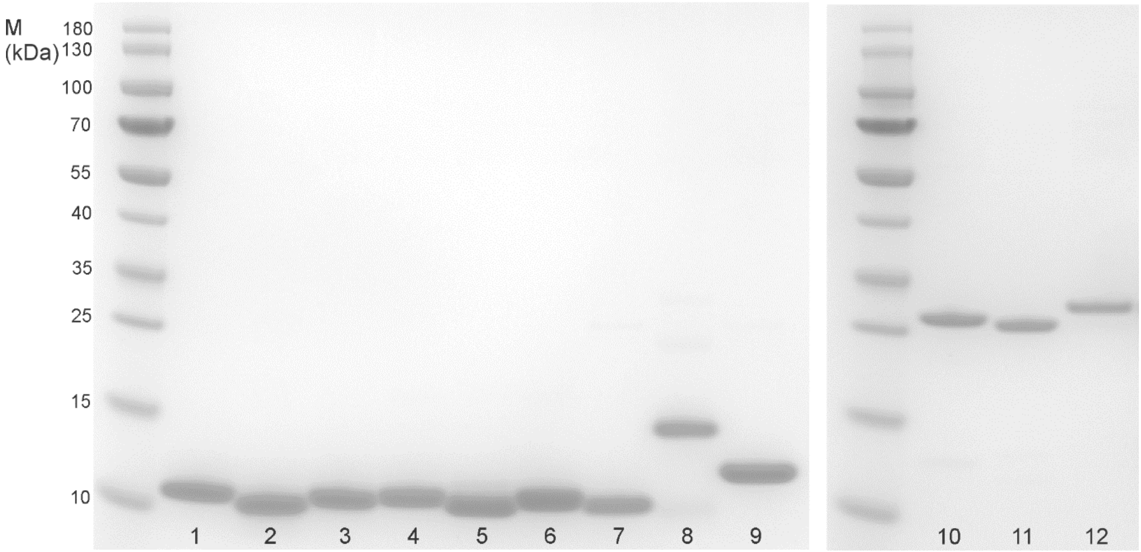

Supplementary Figure 6. SDS-PAGE analysis of purified recombinant proteins used in this study. SDS-PAGE analysis of gp7 protein of GIL01 (lane 1), tectiviral gp7 homologs from prophages found in *B. cereus* VD184, *B. thuringiensis* serovar *aizawai*, *B. cereus* HuB13-1, *B. mycoides* VDm034 phage Lima, *B. anthracis* phage AP50, *B. cereus* AND1284 phage Sato (lanes 2 through 7, respectively), gp7-like proteins M1QNS5 from *B.* and HU from *S. aureus* (lanes 8 and 9, respectively) and LexA proteins from *B. thuringiensis*, *E. coli* and *S. aureus* (lanes 10 through 12, respectively). 2µg of each protein and PageRuler™ prestained protein ladder (Thermo Scientific™) were resolved on SurePAGE™ 4 to 20% bis-Tris protein gels (GenScript) and stained with SimplyBlue SafeStain (Invitrogen).

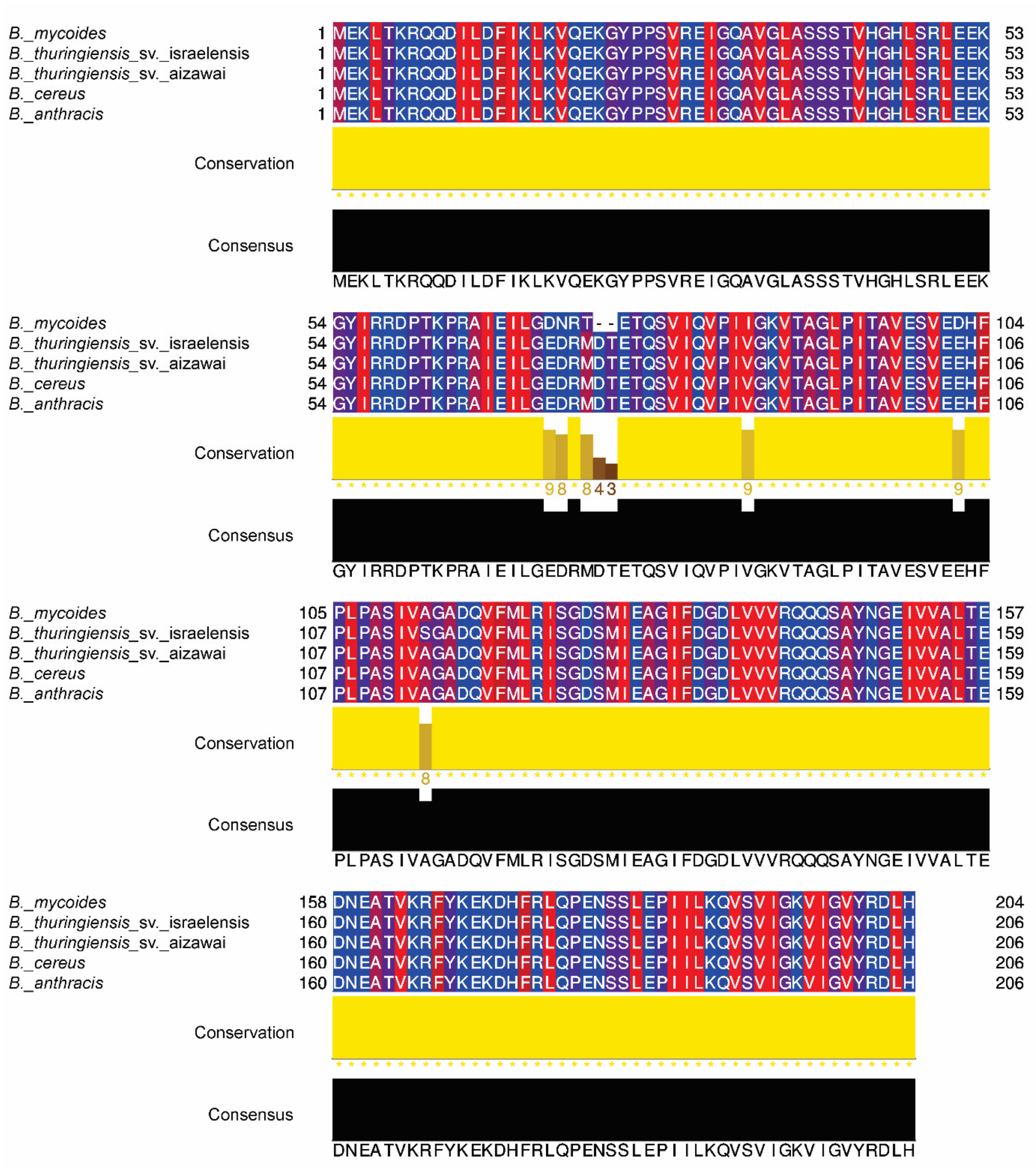

Supplementary Figure 7: **Alignment of amino-acid sequences of *Bacillus* spp. LexA proteins.** LexA protein sequences of *B. thuringiensis* sv. *israelensis* (NCBI Protein ID, QJU63523.1), *B. thuringiensis* sv. *aizawai* (NCBI Protein ID, OIX17173.1), *B. cereus* (UniProt ID, Q81A92), *B. anthracis* (UniProt ID, Q81Y06), and *B. mycoides* (UniProt ID, A9VPX5) were used. The alignment was performed with Clustal Omega and visualized using Jalview. Amino acids are color coded according to the hydrophobicity scale, with red indicating hydrophobic residues and blue indicating hydrophilic residues.

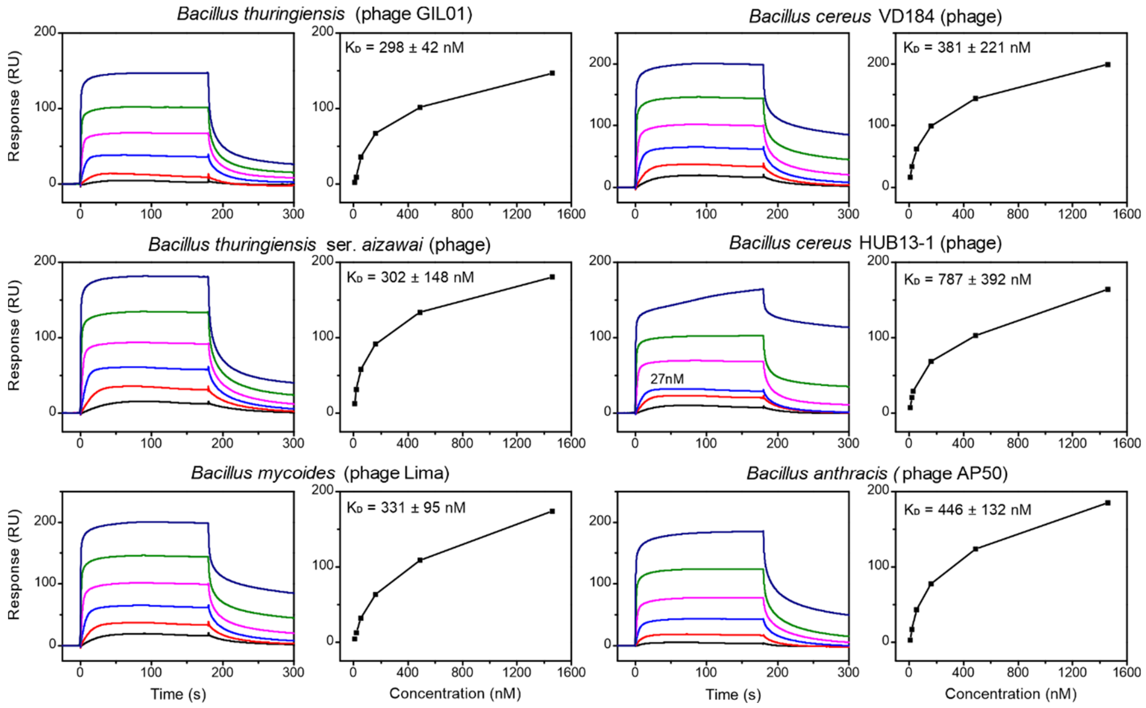

Supplementary Figure 8. **Surface plasmon resonance sensorgrams of the interactions of gp7 and gp7 homologs with *S. aureus* LexA.** Gp7 or its homologs were injected at three-fold dilutions (6-1458 nM) over CM5-chip-immobilized LexA, for 180 s at a flow rate of 30 µL min^{-1}. Apparent equilibrium dissociation constants (K_D) are expressed as means ±standard deviations of two titrations of each analyte. Gp7 and gp7 homolog source organisms and phage names are indicated above the sensorgrams.

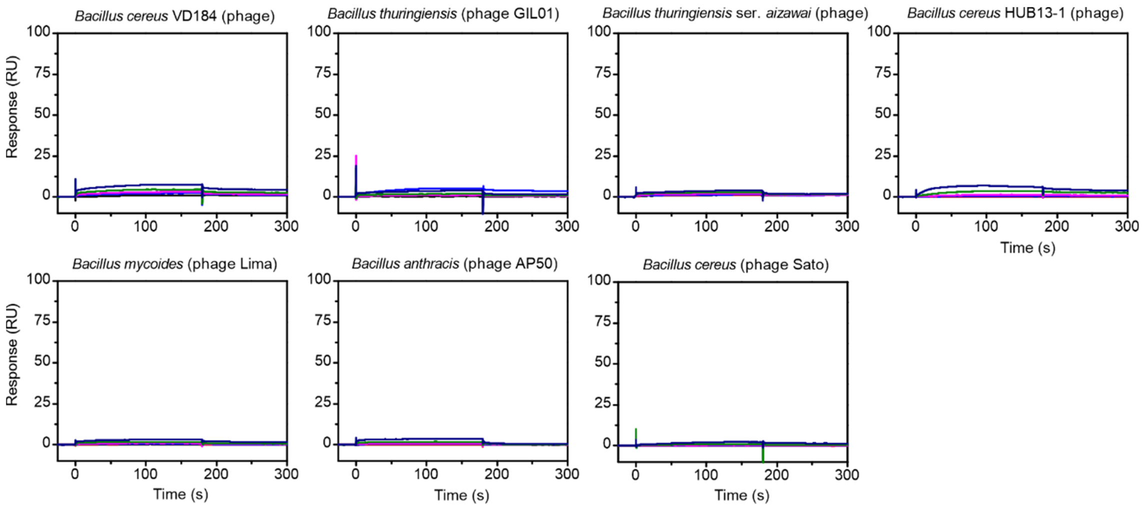

Supplementary Figure 9. **Surface plasmon resonance sensorgrams of the interactions of gp7 and gp7 homologs with the *E. coli* LexA.** Gp7 or its homologs were injected at three-fold dilutions (6-1458 nM) over CM5-chip-immobilized LexA, for 180 s at a flow rate of 30 µL min^{-1}. Gp7 and gp7 homolog source organisms and phage names are indicated above the sensorgrams. Experiments were performed in duplicate and representative sensorgrams are shown.

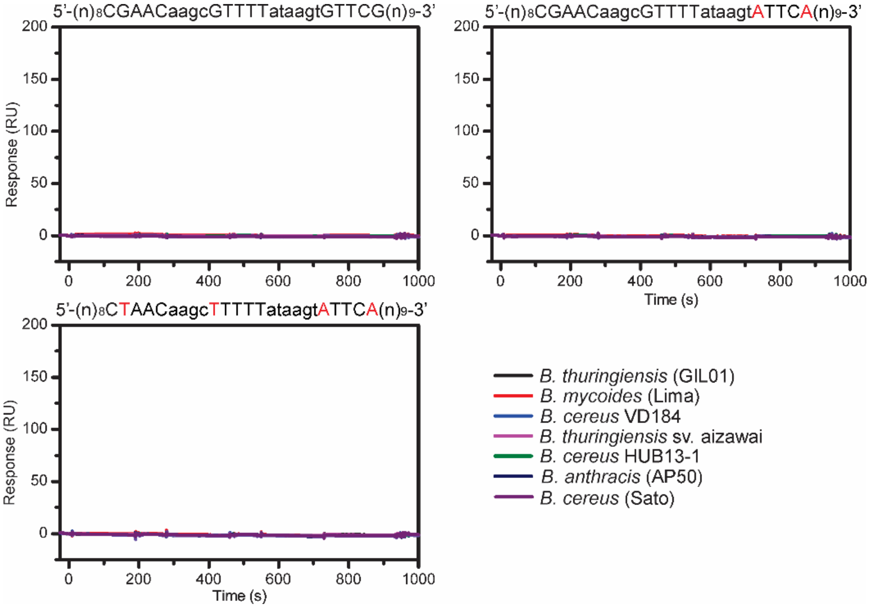

Supplementary Figure 10. Surface plasmon resonance sensorgrams of the interactions of gp7 and gp7 homologs with LexA operators found at the *P1* promoter region of bacteriophage GIL01. Sequences of DNA fragments used are shown above the diagrams, with important sites for LexA binding shown in capital letters, and changed nucleotides shown in red. Gp7 and gp7 homologs (200 nM) were injected over the selected DNAs immobilized on the chip at 50 µL min^{-1}. Experiments were performed in duplicate and representative sensorgrams are shown.

Supplementary Table 1. Strains and plasmids used in this study.

Strain/plasmid		Relevant features	Source/reference
**Strains**	**Ser./Subsp.**		
B. thuringiensis GBJ002	*israelensis*	Derivative of strain 4Q7. Chromosomal resistance to nalidixic acid (Nal^R). Removed were all the plasmids except for pBtic235. Host for GIL01.	[6]
B. thuringiensis GBJ002(GIL01)	*israelensis*	Derivative of strain GBJ002. GIL01 and pBtic235 lysogen.	[7]
B. thuringiensis HER1410	*thuringiensis*	Host for GIL01 and pBtic235. Used for pBtic235 propagation.	Félix d'Hérelle Center for Bacterial Viruses (Canada)
S. aureus ATCC29213	*aureus*	Reference strain in drug discovery.	American-Type Culture Collection (USA)
E. coli MG1655		Descendant of the original K-12 isolate.	American-Type Culture Collection (USA)
E. coli BL21(DE3)		Strain suitable for transformation and high level protein expression using a T7 RNA polymerase-IPTG induction system.	New England Biolabs, USA
**Plasmids**	**Abbreviation**		
pDG148	pDG	*E. coli-Bacillus* spp. shuttle vector for IPTG-inducible gene expression *in Bacillus subtilis*, Km^R, Ap^R	[8]
pDG148-ORF7	pDG7	A pDG148 derivative, carrying GIL01 ORF7, for IPTG-induced expression of gp7 in *B. thuringiensis*, Km^R, Ap^R.	[7]
pET29b(+) *B. cereus* VD184		A pET29(+) derivative, carrying tectiviral gp7 homolog of *B. cereus* VD184, for IPTG-induced expression in *E. coli*, Km^R.	Twist Bioscience
pET29b(+) *B. thuringiensis* sv. *aizawai*		A pET29(+) derivative, carrying tectiviral gp7 homolog of *B. thuringiensis* sv. *aizawai*, for IPTG-induced expression in *E. coli*, Km^R.	Twist Bioscience
pET29b(+) *B. cereus* HuB13-1		A pET29(+) derivative, carrying tectiviral gp7 homolog of *B. cereus HuB13-1*, for IPTG-induced expression in *E. coli*, Km^R.	Twist Bioscience
pET29b(+) *B. mycoides* VDm034 phage Lima		A pET29(+) derivative, carrying tectiviral gp7 homolog of *B. mycoides* VDm034 phage Lima, for IPTG-induced expression in *E. coli*, Km^R.	Twist Bioscience
pET29b(+) *B. anthracis* phage AP50		A pET29(+) derivative, carrying tectiviral gp7 homolog of *B. anthracis* phage AP50, for IPTG-induced expression in *E. coli*, Km^R.	Twist Bioscience
pET29b(+) *B. cereus* AND1284 phage Sato		A pET29(+) derivative, carrying tectiviral gp7 homolog of *B. cereus* AND1284 phage Sato, for IPTG-induced expression in *E. coli*, Km^R.	Twist Bioscience
pET29b(+) M1QNS5		A pET29(+) derivative, carrying putative gp7-like protein M1QNS5 from *B. thuringiesis*, for IPTG-induced expression in *E. coli*, Km^R.	Twist Bioscience
pET29b(+) HU		A pET29(+) derivative, carrying putative gp7-like protein HU from *S. aureus*, for IPTG-induced expression in *E. coli*, Km^R.	Twist Bioscience

Supplementary Table 2. Oligonucleotides used in this study. The oligonucleotide extension required for annealing to the surface plasmon resonance chip immobilized S1 primer is double underlined, important bases for LexA binding are underlined, and substituted nucleotides are shown in bold. 'TEGBio' denotes the tetraethylene glycol spacer arm with biotin tag.

Primer name	Primer sequence 5' -3'
P1-P2_wt_long	GTTACTACTCGAGCGTTAAAAAACGAACAAGCGTTTTATAAGTGTTCGGTTTTTGTA
P1-P2_wt_short	TACAAAAACCGAACACTTATAAAACGCTTGTTCGTTTTTTAA
P1-P2_m1_long	GTTACTACTCGAGCGTTAAAAAACGAACAAGCGTTTTATAAGT**A**TTC**A**GTTTTTGTA
P1-P2_m1_short	TACAAAAAC**T**GAA**T**ACTTATAAAACGCTTGTTCGTTTTTTAA
P1-P2_m2_long	GTTACTACTCGAGCGTTAAAAAAC**T**AACAAGC**T**TTTTATAAGT**A**TTC**A**GTTTTTGTA
P1-P2_m2_short	TACAAAAAC**T**GAA**T**ACTTATAAAA**A**GCTTGTT**A**GTTTTTTAA
pBtic235_long	GTTACTACTCGAGCGATATAATAAGAACATGCGTTCGTGAATTGA
pBtic235_short	TCAATTCACGAACGCATGTTCTTATTATAT
S1	CGCTCGAGTAGTAAC-TEGBio
qPCR_orf7 F	GCGTGACAAATTGCTCGACT
qPCR_orf7 R	AGTCATCCTTCTTCCCCTCCA
qPCR_tufA F	AAATCGACGCTGCTCCAGAA
qPCR_tufA R	CATAGTCAGCGTGACCTGGG

Supplementary References

1. Caveney, N. A. *et al.* Structural insights into bacteriophage GIL01 gp7 inhibition of host LexA repressor. *Structure* **27**, 1094-1102.e4 (2019).
2. Fornelos, N. *et al.* Bacteriophage GIL01 gp7 interacts with host LexA repressor to enhance DNA binding and inhibit RecA-mediated auto-cleavage. *Nucleic Acids Res.* **43**, 7315–7329 (2015).
3. Baebler, Š., Svalina, M., Petek, M., Stare, K., Rotter, A., Pompe-Novak, M., & Gruden, K. quantGenius: implementation of a decision support system for qPCR-based gene quantification. *BMC bioinformatics*, **18**(1), 1-11 (2017).
4. Gillis, A., Guo, S., Bolotin, A., Makart, L., Sorokin, A., & Mahillon, J. Detection of the cryptic prophage-like molecule pBtic235 in *Bacillus thuringiensis* subsp. *israelensis. Res. Microbiol.* **168**, 319–330 (2017).
5. Sullivan, M. J., Petty, N. K. & Beatson, S. A. Easyfig: A genome comparison visualizer. *Bioinformatics* **27**, 1009–1010 (2011).
6. Jensen, G. B., Andrup, L., Wilcks, A., Smidt, L. & Poulsen, O. M. The aggregation-mediated conjugation system of *Bacillus thuringiensis* subsp. *israelensis*: Host range and kinetics of transfer. *Curr. Microbiol.* **33**, 228–236 (1996).
7. Fornelos, N., Bamford, J. K. & Mahillon, J. Phage-borne factors and host LexA regulate the lytic switch in phage GIL01. *J Bacteriol* **193**, 6008–6019 (2011).
8. Joseph, P., Fantino, J. R., Herbaud, M. L. & Denizot, F. Rapid orientated cloning in a shuttle vector allowing modulated gene expression in *Bacillus subtilis*. *FEMS Microbiol. Lett.* **205**, 91–7 (2001).
