# Supplementary material for: A small bacteriophage protein determines the hierarchy over co-residential jumbo phage in Bacillus thuringiensis serovar israelensis
Source: Commun Biol. 2022 Nov 24;5:1286. doi: 10.1038/s42003-022-04238-3 (PMC9700832; doi:10.1038/s42003-022-04238-3)
Supplement: Supplementary file 3 — Description of Additional Supplementary Files [file 42003_2022_4238_MOESM3_ESM.pdf]

Description of Additional Supplementary Files

File name: Supplementary Data 1

Description: : List of differentially expressed genes in MMC-treated versus untreated GBJ002.

File name: Supplementary Data 2

Description: List of differentially expressed genes of MMC-treated strains GBJ002(GIL01) versus GBJ002.

File name: Supplementary Data 3

Description: List of differentially expressed genes of MMC-untreated strains GBJ002(GIL01) versus GBJ002.

File name: Supplementary Data 4

Description: List of differentially expressed genes in MMC-treated versus untreated GBJ002(GIL01).

File name: Supplementary Data 5

Description: List of differentially expressed genes in IPTG-induced and MMC-treated GBJ002(pDG7) versus GBJ002(pDG).

File name: Supplementary Data 6

Description: Protein candidates identified by mass spectrometry that bound to the LexA repressor of Escherichia coli or Staphylococcus aureus.

File name: Supplementary Data 7

Description: The source data behind the graphs in the paper
