# Supplementary material for: A small bacteriophage protein determines the hierarchy over co-residential jumbo phage in Bacillus thuringiensis serovar israelensis
Source: Commun Biol. 2022 Nov 24;5:1286. doi: 10.1038/s42003-022-04238-3 (PMC9700832; doi:10.1038/s42003-022-04238-3)
Supplement: Supplementary file 11 — Reporting Summary [file 42003_2022_4238_MOESM11_ESM.pdf]

nature portfolio

Corresponding author(s): Matej Butala

Last updated by author(s): Sep 16, 2022

Reporting Summary

Nature Portfolio wishes to improve the reproducibility of the work that we publish. This form provides structure for consistency and transparency in reporting. For further information on Nature Portfolio policies, see our Editorial Policies and the Editorial Policy Checklist.

Statistics

For all statistical analyses, confirm that the following items are present in the figure legend, table legend, main text, or Methods section.

n/a	Confirmed	
☐	☒	The exact sample size (*n*) for each experimental group/condition, given as a discrete number and unit of measurement
☐	☒	A statement on whether measurements were taken from distinct samples or whether the same sample was measured repeatedly
☐	☒	The statistical test(s) used AND whether they are one- or two-sided<br>*Only common tests should be described solely by name; describe more complex techniques in the Methods section.*
☐	☒	A description of all covariates tested
☐	☒	A description of any assumptions or corrections, such as tests of normality and adjustment for multiple comparisons
☐	☒	A full description of the statistical parameters including central tendency (e.g. means) or other basic estimates (e.g. regression coefficient) AND variation (e.g. standard deviation) or associated estimates of uncertainty (e.g. confidence intervals)
☐	☒	For null hypothesis testing, the test statistic (e.g. *F*, *t*, *r*) with confidence intervals, effect sizes, degrees of freedom and *P* value noted<br>*Give P values as exact values whenever suitable.*
☒	☐	For Bayesian analysis, information on the choice of priors and Markov chain Monte Carlo settings
☐	☒	For hierarchical and complex designs, identification of the appropriate level for tests and full reporting of outcomes
☐	☒	Estimates of effect sizes (e.g. Cohen's *d*, Pearson's *r*), indicating how they were calculated

Our web collection on statistics for biologists contains articles on many of the points above.

Software and code

Policy information about availability of computer code

Data collection	Biacore T200 control software 3.2 was used to collect the surface plasmon resonance spectrometry data. Illumina NovaSeq control software v1.7 was used to collect transcriptomic data. Bacteriophage pBtic235 was visualized by transmission electron microscopy using the CM100 and software Digital Micrograph v2.11. G:Box software was used to collect images of agarose plates, SDS PAGE gels.
Data analysis	Biacore T200 Evaluation Software 3.2 was used for the analysis of the surface plasmon resonance data.<br>For the transcriptomic analysis, we designed a custom Snakemake workflow available at https://github.com/AnzeLovse/mag. Genome coverage was calculated using the genomeCoverageBed command from Bedtools (2.30.0). Coverage was normalized and then visualized using the ggplot2 R package (3.3.6). Graphs were drawn in Origin 2020.

For manuscripts utilizing custom algorithms or software that are central to the research but not yet described in published literature, software must be made available to editors and reviewers. We strongly encourage code deposition in a community repository (e.g. GitHub). See the Nature Portfolio guidelines for submitting code & software for further information.

Data

Policy information about availability of data

All manuscripts must include a data availability statement. This statement should provide the following information, where applicable:

- Accession codes, unique identifiers, or web links for publicly available datasets
- A description of any restrictions on data availability
- For clinical datasets or third party data, please ensure that the statement adheres to our policy

Raw RNA sequence data files have been deposited under the Bioproject with accession number PRJNA780269 (https://dataview.ncbi.nlm.nih.gov/object/PRJNA780269?reviewer=8t7iaetkh5vr1mfbpktbalfqrb) and BioSample accession numbers: SAMN28961381 (GBJ002/GIL01_MMC), SAMN28961382(GBJ002/GIL01),

SAMN28961383 (GBJ002_MMC), SAMN28961384(GBJ002), SAMN23134701 (GBJ002_pDG), SAMN23134702 (GBJ002_pDG7), SAMN23134703 (GBJ002_pDG_MMC), and SAMN23134704 (GBJ002_pDG7_MMC). Raw genome sequencing data files have been deposited with BioSample accession numbers: SAMN23134705 (pBtic235_WGS), SAMN30884991 (GBJ396_WGS), and SAMN30884992 (GBJ002_WGS).

Field-specific reporting

Please select the one below that is the best fit for your research. If you are not sure, read the appropriate sections before making your selection.

☒ Life sciences ☐ Behavioural & social sciences ☐ Ecological, evolutionary & environmental sciences

For a reference copy of the document with all sections, see nature.com/documents/nr-reporting-summary-flat.pdf

Life sciences study design

All studies must disclose on these points even when the disclosure is negative.

Sample size	The sample size of transcriptomic experiments, protein-DNA SPR interaction experiments was chosen in consistency with many other publications that used similar number of biological replicates (e.g. PMID: 33051375, 31056420). The size of samples was not determined by a statistical method. The TEM micrograph of bacteriophage pBtic235 is a representative of more than 20 virions visualized. Full information on sample size is in figure legend and methods.
Data exclusions	For consistency, we clearly specify in the figure legend when IPTG and when mitomycin C were added to the cultures and when the samples were collected for RNA sequencing analysis. All attempts of transcription analysis were successful. In Figures 2B, 3B, 3C, 3D, 4B, and 4C we show representative results.
Replication	All attempts of replication were successful. The number of times an experimental finding was reproduced is indicated in the associated figure legend for each experiment shown.
Randomization	No randomization methods were used because this was not applicable for our experiments. n/a
Blinding	n/a

Reporting for specific materials, systems and methods

We require information from authors about some types of materials, experimental systems and methods used in many studies. Here, indicate whether each material, system or method listed is relevant to your study. If you are not sure if a list item applies to your research, read the appropriate section before selecting a response.

Materials & experimental systems

n/a	Involved in the study	
☒	☐	Antibodies
☒	☐	Eukaryotic cell lines
☒	☐	Palaeontology and archaeology
☒	☐	Animals and other organisms
☒	☐	Human research participants
☒	☐	Clinical data
☒	☐	Dual use research of concern

Methods

n/a	Involved in the study	
☒	☐	ChIP-seq
☒	☐	Flow cytometry
☒	☐	MRI-based neuroimaging
